# Designing highly emissive over-1000 nm near-infrared fluorescent dye-loaded polystyrene-based nanoparticles for *in vivo* deep imaging†

**DOI:** 10.1039/d1ra01040a

**Published:** 2021-05-25

**Authors:** Yuichi Ueya, Masakazu Umezawa, Eiji Takamoto, Moe Yoshida, Hisanori Kobayashi, Masao Kamimura, Kohei Soga

**Affiliations:** Tsukuba Research Laboratories, JSR Corporation 25 Miyukigaoka Tsukuba Ibaraki 305-0841 Japan; Department of Materials Science and Technology, Faculty of Advanced Engineering, Tokyo University of Science 6-3-1 Niijuku Katsushika Tokyo 125-8585 Japan masa-ume@rs.tus.ac.jp mail@ksoga.com

## Abstract

Polystyrene-based nanoparticles (PSt NPs) prepared by emulsion polymerization are promising organic matrices for encapsulating over-thousand-nanometer near-infrared (OTN-NIR) fluorescent dyes, such as thiopyrilium IR-1061, for OTN-NIR dynamic live imaging. Herein, we propose an effective approach to obtain highly emissive OTN-NIR fluorescent PSt NPs (OTN-PSt NPs) in which the polarity of the PSt NPs was adjusted by changing the monomer ratio (styrene to acrylic acid) in the PSt NPs and the dimethyl sulfoxide concentration in the IR-1061 loading process. Moreover, OTN-PSt NPs covalently modified with poly(ethylene glycol) (PEG) (OTN-PSt-PEG NPs) showed high dispersion stability under physiological conditions and minimal cytotoxicity. Notably, the optimized OTN-PSt-PEG NPs were effective in the dynamic live imaging of mice. This methodology is expected to facilitate the design of certain polar thiopyrilium dye-loaded OTN-NIR fluorescent imaging probes with high emissivity.

## Abstract 2

By changing the ratio of acrylic acid to styrene, the loading amount of fluorescent dye can be increased and the optical properties of the resulting bioimaging probe can be improved.

## Graphical abstract

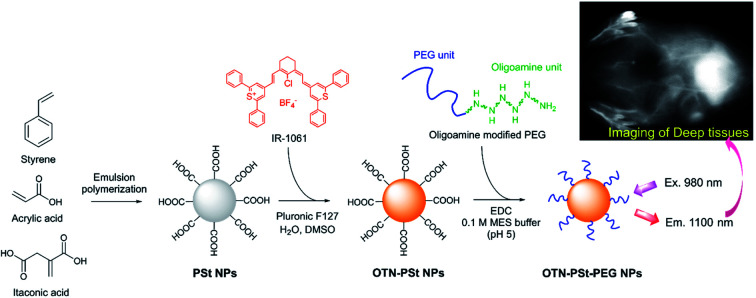

## Introduction

Dynamic live imaging of biological structures and phenomena in deep tissues has been a fundamental challenge in biomedical research.^1–3^ In the last decade, there has been growing interest in over-thousand-nanometer near-infrared (OTN-NIR) fluorescence imaging to enable live and dynamic observations of deep tissues.^4–6^ Research on OTN-NIR fluorescence imaging has been pursued for both the development of systems for high-precision and multidimensional imaging as well as the development of functional fluorescent probes.^7^ Various nanomaterials have been developed as OTN-NIR fluorophores, such as rare-earth-doped ceramic nanoparticles,^8–12^ carbon nanotubes,^13–17^ quantum dots,^18,19^ and organic dye-based phosphors including novel organic dyes,^20–23^ dye molecular aggregations,^24–28^ dye-encapsulated micelles and soft nanoparticles (NPs),^29–32,35,36,44^ dye-conjugated peptides,^33^ and dye-loaded silicaa NPs.^34^ Fluorescent probes by encapsulating dyes in biocompatible molecules using hyaluronic acid^45^ and apoferritin^46^ were also reported for cancer imaging and photothermal therapy. Organic dye-based phosphors have been the focus of some research because of their low toxicity in clinical applications. Since several well-known and commercially available OTN-NIR fluorescent dyes, such as IR-26, IR-1048, and IR-1061, are water-insoluble, nanometer-scale polymer micelles based on amphiphilic polymers have been proposed as encapsulating matrices.^29,31^ However, because self-assembled polymer micelles are stabilized with weak interactive forces, they are prone to *in vivo* decomposition or encapsulated dye leakage. Controlling micellar stability has been a major unsolved issue, which has encumbered further applications of micelles as probes.

Recently, we reported a new OTN-NIR dye-loaded and poly(ethylene glycol) (PEG)-modified polystyrene-based NP (OTN-PSt-PEG NP) with demonstrative *in vivo* imaging data.^37^ PSt NPs, which are latex emulsions prepared by emulsion polymerization, have been widely used as the solid phase of *in vitro* latex agglutination assays because they are robust and do not decompose in biological fluids.^38,39^ Therefore, PSt NPs are considered preferred matrices for *in vivo* environments. Furthermore, emulsion polymerization produces polymer particles with sizes ranging from sub-100 nm to several 100 nm.^40^ Notably, sub-100 nm NPs show high retention in the blood circulation,^41^ which is required for some *in vivo* applications such as cancer imaging.

IR-1061, a commercially available OTN-NIR fluorescent dye, can be introduced into PSt NPs by swelling–diffusion processes.^42^ Moreover, IR-1061 is a polar and hydrophobic molecule and causes aggregation and fluorescent quenching in environments with mismatched polarities (Umezawa *et al.* unpublished data). Therefore, it is important the polarities of IR-1061 and the PSt NP core are matched. However, the effects of tuning factors of the preparation process on the properties of obtained NPs were not clarified. In this study, we investigated the conditions under which highly emissive OTN-PSt NPs can be obtained by optimizing the polarity of the PSt NPs, the organic solvent to water ratio in the swelling–diffusion process, and the IR-1061 to PSt NPs ratio. Furthermore, the applicability of the OTN-PSt-PEG NP as a photoluminescent probe was confirmed with the results of stability tests in physiological aqueous environments, cytotoxicity assessments, and *in vivo* OTN-NIR fluorescent imaging.

## Experimental

### Materials

Styrene was purchased from Chuo Kasei Co., Ltd (Osaka, Japan). Acrylic acid solution (80%) was purchased from Toagosei Co., Ltd (Tokyo, Japan). Itaconic acid was purchased from Fuso Chemical Co., Ltd (Osaka, Japan). Sodium dodecylbenzene sulfonate was purchased from Kao Co. (Tokyo, Japan). Potassium persulfate was purchased from ADEKA Co. (Tokyo, Japan). IR-1061 and pluronic F127 were purchased from Sigma-Aldrich (St. Louis, MO, USA). Dimethyl sulfoxide (DMSO), Dulbecco's phosphate buffered saline (D-PBS), D-MEM (high glucose) with l-glutamine and phenol red, and penicillin–streptomycin–amphotericin B suspension (×100) (antibiotic–antimycotic solution) were purchased from Fujifilm Wako Pure Chemical Co. (Osaka, Japan). Methoxy PEG modified with oligoamine at one end, Blockmaster™ CE210, was purchased from JSR Life Sciences Co. (Tokyo, Japan). 1-Ethyl-3-(3-dimethylaminopropyl)carbodiimide hydrochloride (EDC) and lactate dehydrogenase (LDH) assay kit-WST were purchased from Dojindo Laboratories (Kumamoto, Japan). The mouse fibroblast cell line NIH-3T3 was purchased from ATCC (Manassas, VA, USA). Fetal bovine serum (FBS) was purchased from Cytiva (Mariborough, MA, USA). All the reagents were used without further purification.

### Preparation of OTN-PSt NPs

PSt NPs were synthesized by conventional oil-in-water emulsion polymerization. A mixture of 403 mL of water, 4.1 g (11.8 mmol) of sodium dodecylbenzenesulfonate, 78.0 g (749 mmol) of styrene, 20.9 g (288 mmol) of acrylic acid, and 1.10 g (8.49 mmol) of itaconic acid was added into a 500 mL separable flask and kept at 80 °C under a nitrogen atmosphere for 30 min. Polymerization started when 0.301 g (1.11 mmol) of potassium persulfate in 8.6 mL of water was added into the flask. After the reaction at 80 °C for 5 h, the obtained PSt NPs were purified by dialysis (MWCO 14 000, Sekisui Medical Co. Ltd, Tokyo, Japan) to remove unreacted components and to replace the dispersion medium with water. Next, 0.25–4 mg of IR-1061, 1.0 g of PSt NPs, and 140 mg of pluronic F127 were mixed in 100 mL of an aqueous solution of 5–50% DMSO by stirring for 10 min to introduce IR-1061 into the PSt NPs. The obtained OTN-PSt NPs were purified by dialysis (MWCO 50 000, Spectrum Chemical Mfg Co., NJ, USA) to replace the dispersion medium with water.

### Preparation of OTN-PSt-PEG NPs

**Fig. 1:**
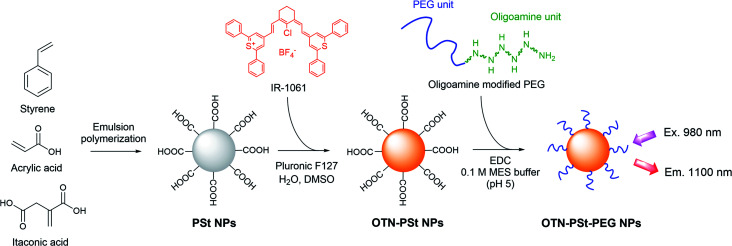
Schematic illustration of preparation of OTN-PSt-PEG NPs.

Into a mixture of 3 mL of 5% (w/w) OTN-PSt NP dispersion and 3.75 mL of Blockmaster™ CE210, 1.5 mL of 1% (w/w) EDC in MES buffer (pH 5) was added. The mixture was allowed to react for 2 h at 20 °C. The obtained OTN-PSt-PEG NPs (Fig. 1) were purified by dialysis (MWCO 300 000, Spectrum Chemical Mfg) to remove the unreacted components and to replace the dispersion medium with water.

### Characterization of OTN-PSt NPs and OTN-PSt-PEG NPs

The size of the NPs was analyzed by dynamic light scattering (DLS; Nanotrac UPA EX150, MicrotracBEL Co., Osaka, Japan) and scanning electron microscopy (SEM; SU8020, Hitachi Ltd, Tokyo, Japan). The absorption spectra of the NP samples were measured using an ultraviolet-visible-NIR spectrophotometer (V-770, JASCO Co., Tokyo, Japan). The loading concentration of IR-1061 was calculated by the relation between the concentration and its optical absorbance.^32^ The emission spectra of the NP samples were measured using a spectrofluorometer (Fluorolog-3, Horiba Ltd, Kyoto, Japan) and a spectrometer (NIR-256-1.7; Avantes, Apeldoorn, Netherlands) equipped with a fiber-coupled diode (SP-976-5-1015-7; Laser Components GmbH, Olching, Germany) as the 980 nm excitation source.

### Cytotoxicity of OTN-PEG-PSt NPs

The NIH-3T3 cells were cultured in D-MEM containing 10% FBS and 1% penicillin–streptomycin at 37 °C in a humidified atmosphere containing 5% CO_2_. The cytotoxicity of the NP samples was assessed in 3T3 cells by the standard LDH assay according to the manufacturer's instructions. Briefly, the cells were seeded in 96-well microplates with 1.5 × 10^5^ cells per well and allowed to adhere for 24 h prior to the assay. The cells were exposed to various concentrations (0.2–20 mg mL^−1^) of OTN-PSt NPs or OTN-PSt-PEG NPs for 21 h at 37 °C, followed by washing with D-PBS, after which the cell viability was evaluated using the LDH assay. Absorption was measured at 450 nm in a microplate reader (Cytation 5; BioTek Instruments, Inc., VT, USA). Cytotoxicity was determined by the LDH production normalized by the positive control cells treated with the lysis buffer in the kit.

### OTN-NIR fluorescence *in vivo* imaging

All animal care and experiment procedures were performed in accordance with the Guidelines for Care and Use of Laboratory Animals of Tokyo University of Science under approval by the Animal Ethic Committee of Tokyo University of Science. Three-week-old male ICR mice were purchased from Japan SLC (Hamamatsu, Japan). Before *in vivo* imaging experiments, to lower the body level of phosphorescent alfalfa, the mice were fed the AIN-76A diet (Research Diets, New Brunswick, NJ, USA) for 20 days. After this, the mice were anesthetized, and their hair was removed to avoid light scattering. Following this, 100 μL of OTN-PSt-PEG NP dispersion in D-PBS (20 mg mL^−1^) was injected into the tail vein. The OTN-NIR fluorescence images were observed using an OTN-NIR fluorescence *in vivo* imaging system (SAI-1000; Shimadzu Co., Kyoto, Japan).

## Results and discussion

### Optimization of OTN-PSt NPs

**Fig. 2:**
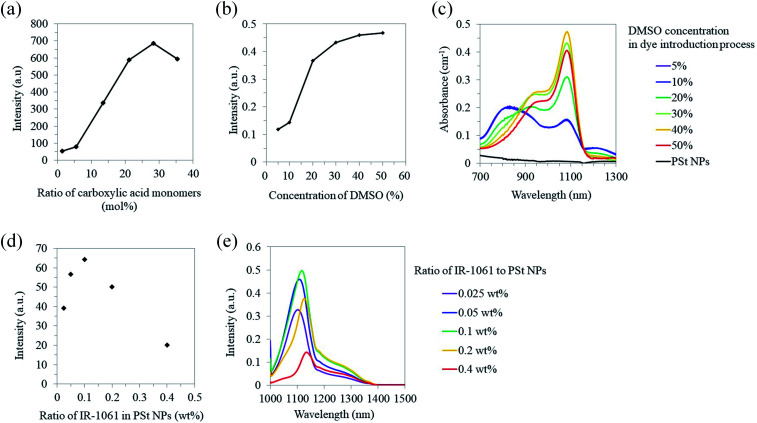
Optical properties of OTN-PSt NPs. (a) Photoluminescent intensity at 1100 nm of the OTN-PSt NPs (5 mg mL^−1^ in water) prepared with different ratios of styrene to acrylic acid. (b) Photoluminescent intensity at 1100 nm of the OTN-PSt NPs (5 mg mL^−1^ in water) prepared with different DMSO concentrations. (c) Absorption spectra of the OTN-PSt NPs (5 mg mL^−1^ in water). (d) Photoluminescent intensity integrated over 1000–1300 nm and (e) spectra of the OTN-PSt NPs (5 mg mL^−1^ in water) prepared with different dye contents. Fluorescence was collected by the spectrometer under 980 nm laser excitation (4.2 W).

We designed PSt NPs copolymerized with acrylic acid to bind aminated PEG *via* amide bonding on the NP surface (Fig. 1). In addition, we prepared 6 types of PSt NPs with different monomer ratios using 72.0–79.0 g (691–951 mmol) of styrene and 27.0–0.94 g (366–13 mmol) of acrylic acid (Table 1) to investigate the effect of PSt NP polarity on the resulting fluorescence. The size of each PSt NP was approximately 40–50 nm (Table 1 and ESI Fig. S1†) and considered to be preferable for high retention in the blood circulation.^41^ For the PSt NPs with the same IR-1061 loading, the photoluminescent intensity of the OTN-PSt NPs was the highest when the styrene : acrylic acid ratio was 72 : 28, which was ∼12 times higher than that of the sample prepared with a monomer ratio of 99 : 1 (Fig. 2a). PSt is composed of hydrocarbons with a highly hydrophobic phenyl group. Similarly, IR-1061 is a hydrophobic dye with an intramolecularly polarized chemical structure. Introducing acrylic acid into PSt polarizes the molecule and improves the affinity of the resulting hydrophobic NPs for IR-1061. As a result, increasing the acrylic acid content increased the amount of dye incorporated in the PSt NPs and the fluorescent intensity of OTN-PSt (Fig. 2a). However, the absorption spectra suggested that even in the PSt NPs showing high emission intensities, the IR-1061 dye in the PSt NPs showed the typical absorption spectra influenced strongly by water (ESI Fig. S2 and S3†), of which the polar O–H bond often quenches OTN-NIR luminophore.^43^ Moreover, IR-1061 was concentrated on the surface *via* insufficient infiltration into the core of PSt NPs which were prepared in less DMSO. In this case, because of the water-insolubility of IR-1061, it may form like H-aggregates of cyanine dyes^49^ and thus quenched possibly due to non-emissive intramolecular energy transfer.

**Table 1:** Styrene to acrylic acid monomer ratios investigated for preparing OTN-PSt NPs

Styrene (mol%)	Acrylic acid monomers (mol%)	Size (nm)
65	35	52
72	28	48
79	21	44
86	14	46
94	6	41
99	1	48

The NPs were synthesized with 72.0–79.0 g (691–951 mmol) of styrene and 27.0–0.94 g (366–13 mmol) of acrylic acid *via* the method described in Preparation of OTN-PSt NPs. The listed size is the hydrodynamic diameter of the NPs dispersed in water determined by dynamic light scattering. The size distribution of each sample is shown in ESI Fig. S1.†

To introduce IR-1061 into the core of the PSt NPs, we increased DMSO concentration from 5% to 50% in the IR-1061 loading process with the PSt NPs prepared with a monomer ratio of 28 mol% acrylic acid. The photoluminescent intensity increased with higher DMSO concentrations, especially within 20–40%, although the DMSO effect on intensity saturated at 40% (Fig. 2b). The photoluminescent intensity of the OTN-PSt NPs prepared with 30% DMSO concentration was approximately 3.5 times larger than that of the sample prepared with 5% DMSO. For the PSt NPs prepared with 5–10% DMSO, the absorption peak at 820 nm indicated that IR-1061 adopted the quenched coupled form with water molecules (Fig. 2c), possibly because most of the dye was loaded onto the NP surface by insufficient infiltration into the NP core. This quenching caused by the dye coupling with water molecules was considerably suppressed in the case of the PSt NPs prepared with 30–50% DMSO. Therefore, increasing the DMSO content in the solvent possibly promoted the swelling of the PSt NPs and enhanced the infiltration of IR-1061 into the NP core and the emission intensity of the products. However, the total dye loading was saturated at approximately 0.12% (w/w) in the OTN-PSt NPs. Thus, in the following experiments, the OTN-PSt NPs were prepared with the styrene : acrylic acid ratio of 72 : 28 (mol%) in an aqueous solution of 30% DMSO.

Furthermore, the effect of IR-1061 loading on the photoluminescent intensity of the OTN-PSt NPs was investigated (Fig. 2d). When the ratio of IR-1061 to PSt NP was ≤0.1 wt% in the loading process, the emission intensity of the OTN-PSt NPs increased as the IR-1061 content increased. In contrast, when the IR-1061 to PSt NP ratio was >0.1 wt%, the emission intensity of the OTN-PSt NPs decreased with increasing dye content (Fig. 2d and e). This was probably due to concentration quenching of IR-1061 in the PSt NPs. Therefore, for highly emissive OTN-PSt NPs, the optimum IR-1061 to PSt-NP ratio was 0.1 wt%. The quantum yield of the OTN-PSt-PEG NP synthesized by this optimized protocol was 0.65% (ESI Fig. S4†).

### Stability and safety of optimized OTN-PSt-PEG NPs

**Fig. 3:**
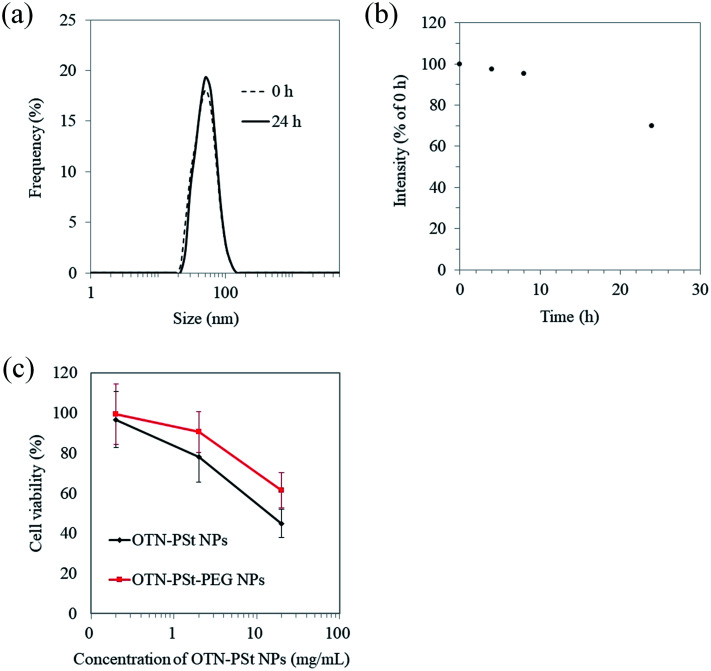
*In vitro* stability of OTN-PSt-PEG NPs. (a) Dispersion stability of OTN-PSt-PEG NPs determined by the hydrodynamic diameter in FBS at 37 °C for 24 h. (b) Stability of the photoluminescent intensity of OTN-PSt-PEG NPs in FBS at 37 °C. (c) Cytotoxicity of OTN-PSt-PEG NPs on cultured 3T3 cells. Measurement sample: OTN-PSt-PEG PSt and OTN-PSt NPs with a styrene to acrylic acid ratio of 72 : 28 (mol%).

The *in vitro* stability of the OTN-PSt-PEG NPs was assessed by analyzing the time-dependent changes in the hydrodynamic diameter and OTN-NIR photoluminescent intensity of the OTN-PSt-PEG NPs dispersed in FBS for 24 h. The diameter of the OTN-PSt-PEG NPs was constant at 45 nm in FBS for 24 h (Fig. 3a). Fig. 3b shows that the OTN-NIR photoluminescent intensity of the OTN-PSt-PEG NPs was constant for 8 h and was maintained at 70% for 24 h in FBS. These results indicate that the OTN-PSt-PEG NPs can maintain their photoluminescence properties without aggregation even in physiological aqueous environments. Compared to the use of non-covalent PEG linking using amphiphilic polymers,^31,47^ the NP design of covalent PEG modification has a potential to provide a choice of which a high stability is expected. However, the luminescence intensity of OTN-PSt-PEG NPs gradually decreased at 37 °C in FBS. Further investigations are needed to control and improve the stability of OTN-PSt PEG NPs. The potential of photodegradation of such organic dyes should also be noted.^48^

To evaluate the safety of the OTN-PSt-PEG NPs for potential biomedical use, the cytotoxicity of the OTN-PSt-PEG NPs was assessed using the LDH assay. The OTN-PSt NPs showed no cytotoxicity at 2 mg mL^−1^ (Fig. 3c). Furthermore, the OTN-PSt-PEG NPs showed lower cytotoxicity than the OTN-PSt NPs, indicating that PEG modification increased the biocompatibility of the PSt NPs.

### OTN-NIR fluorescence *in vivo* imaging

**Fig. 4:**
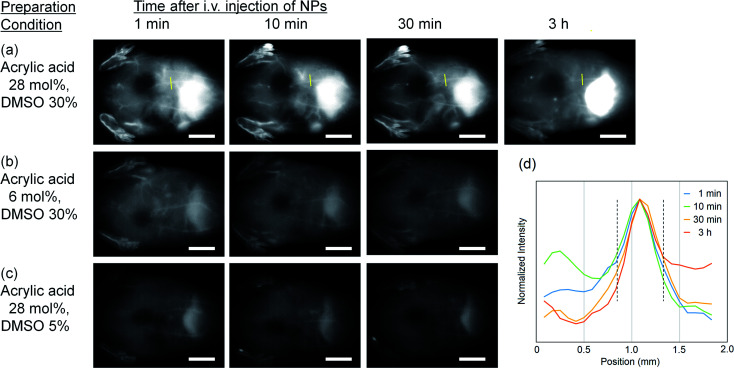
OTN-NIR fluorescence *in vivo* imaging of live mice. The OTN-PSt-PEG NPs (2 mg) dispersed in PBS (0.1 mL) were intravenously injected into 6 week-old male ICR mice. The OTN-NIR fluorescence images were recorded under 980 nm light irradiation (0.4 W cm^−2^) with an integration time of 500 ms. The images show the angiography results of (a) OTN-PSt-PEG NPs prepared with (a) 28 mol% of acrylic acid in PST in 30% DMSO, (b) NPs prepared with less acrylic acid, and (c) NPs prepared in less DMSO as indicated. Scale bars indicate 10 mm. (d) Line profile of the luminescence intensity showing the thickness of blood vessel (indicated by yellow line in (a)) under the skin. The area between the dashed lines indicates a 0.5 mm-thick blood vessel under the skin.

As a demonstration of *in vivo* OTN-NIR fluorescence imaging, we performed angiography of the abdominal blood vessels with the OTN-PSt-PEG NPs in mice. Following the intravenous injection of the OTN-PSt NPs *via* the tail vein and excitation at 980 nm, whole-body blood vessels under the skin were immediately observed with high clarity (Fig. 4). Imaging of the blood vessels was possible even at 3 h after injection, indicating high retention of the OTN-PSt-PEG NPs in the blood circulation. For example, an approximately 0.5 mm-thick blood vessel was observed even at 3 h post-injection as shown in Fig. 4d. These results confirmed that optimizing the monomer ratio in the PSt NPs and the DMSO concentration in the dye-loading process improved the photoluminescence of the resulting NPs for *in vivo* deep imaging. Further, the blood retention for 3 h gave a new option of the design of OTN-NIR fluorescence probes, based on this chemically tuned PSt-based material shown in this paper, for imaging of deep lesion of cancer in preclinical models.

The blood retention followed by their *in vivo* behavior may be controlled by not only PEG coating but also functionalization of PEG, *i.e.*, introduction of functional groups such as amino and carboxyl groups, on the NP surface. Further investigation is needed to clarify the potential effect of surface functionalization of the OTN-PSt-PEG NPs on their *in vivo* behaviors. Not only the surface modification, the choice of the core matrix is also important. For example, the use of biodegradable polymers such as poly(lactic acid) is also interesting for clinical applications and will be reported in next papers.

## Conclusions

In the present study, we demonstrated an optimization method to obtain highly emissive OTN-NIR fluorescent PSt-based NPs containing IR-1061. The photoluminescent intensity of the OTN-PSt NPs changed depending on the monomer ratio (styrene to acrylic acid) in the PSt NPs. Adjusting the polarity of the PSt NPs was an effective approach to increase the loading of the polar dye IR-1061 into the PSt NPs and the resulting emission. Increasing the DMSO concentration from 5% to 30% in the IR-1061 loading process also enhanced the loading of IR-1061 into the PSt NPs, possibly because PSt swelling was promoted. Various fluorescence probes have been already reported by introducing fluorescent dyes into nanoparticle matrix with different *in vivo* stability and degradability. The OTN-PSt NP reported in our current paper add a new option here, and also show that the introduction of acrylic acid into the PSt matrix is important for optimizing the dye loading efficiency. The OTN-PSt-PEG NP, which is OTN-PSt NP covalently modified with biocompatible PEG, showed high dispersion stability in physiological conditions and minimal cytotoxicity. Furthermore, the OTN-PSt-PEG NPs was effective in the whole-body imaging of blood vessels for 3 h after their intravenous injection, in mice. The proposed design approach for the preparation of highly emissive OTN-PSt-PEG NPs for *in vivo* deep imaging is potentially applicable to other polar dye-loaded NPs.

## Author contributions

K. Soga was the main project leader and conceived the overall research idea. Y. Ueya and M. Kamimura designed the components and protocols to develop OTN-PSt-PEG. Y. Ueya, M. Umezawa, M. Yoshida, and H. Kobayashi performed the experiments and data collection. Y. Ueya and E. Takamoto were substantially involved in data analysis. The manuscript was originally drafted by Y. Ueya and M. Umezawa and mainly edited by M. Kamimura and K. Soga. All authors read and approved the final manuscript prior to submission.

## Conflicts of interest

The authors have no conflict of interests to declare related to this manuscript.

## Supplementary Material

RA-011-D1RA01040A-s001
