# Supplementary material for: Designing highly emissive over-1000 nm near-infrared fluorescent dye-loaded polystyrene-based nanoparticles for in vivo deep imaging
Source: RSC Adv. 2021 May 25;11(31):18930–7. doi: 10.1039/d1ra01040a (PMC9033499; doi:10.1039/d1ra01040a)
Supplement: RA-011-D1RA01040A-s001 [file RA-011-D1RA01040A-s001.pdf]

Electronic Supplementary Material (ESI) for RSC Advances.
This journal is © The Royal Society of Chemistry 2021

Supplementary Information

Designing highly emissive over-1000-nm near-infrared fluorescent dye-loaded polystyrene-based nanoparticles for *in vivo* deep imaging

Yuichi Ueya,[a] Masakazu Umezawa,*[,b] Eiji Takamoto,[a] Moe Yoshida,[b] Hisanori Kobayashi,[b] Masao Kamimura [b] and Kohei Soga *[,b]

Tsukuba Research Laboratories, JSR Corporation, 25 Miyukigaoka, Tsukuba, Ibaraki, 305-0841, Japan.
Department of Materials Science and Technology, Faculty of Industrial Science and Technology, Tokyo University of Science, 6-3-1 Niijuku, Katsushika, Tokyo 125-8585, Japan.
*Corresponding to masa-ume@rs.tus.ac.jp (M. Umezawa) and mail@ksoga.com (K. Soga)

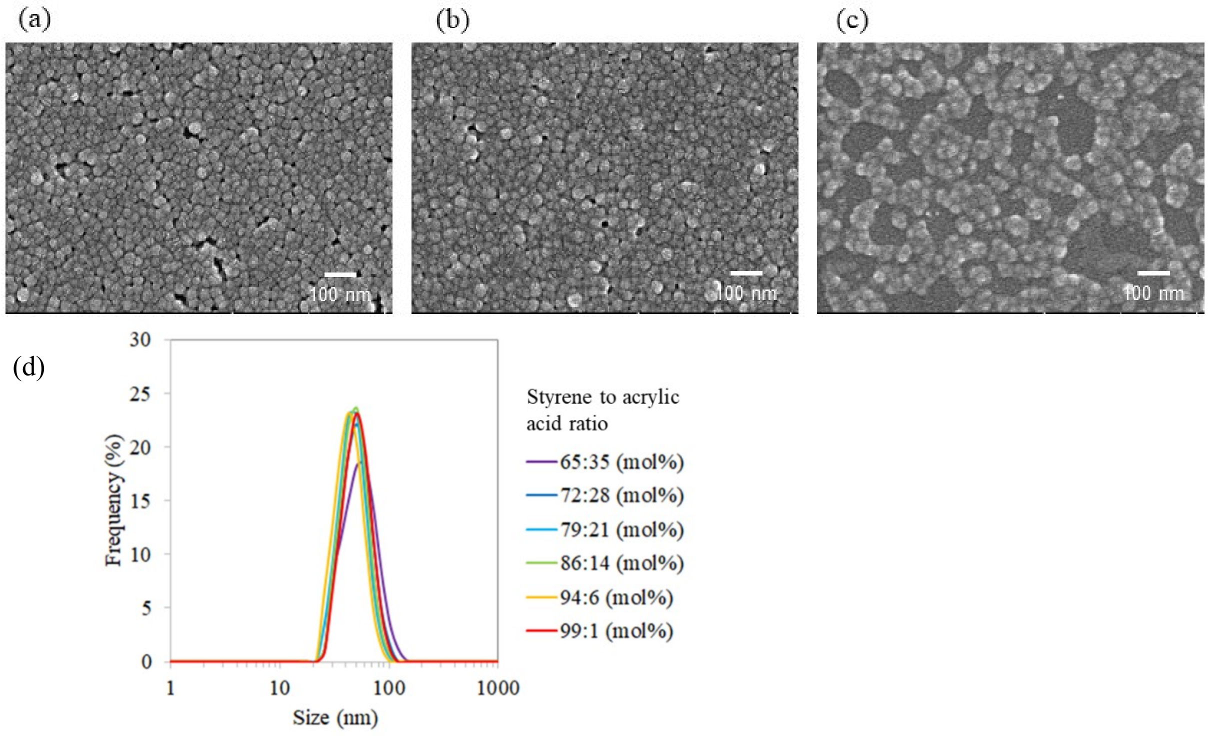

Supplementary Figure S1. Scanning electron microscope (SEM) images and size distributions of NPs. (a) PSt NPs (b) OTN-PSt NPs. (c) OTN-PSt-PEG NPs. All samples were synthesized by styrene and acrylic acid monomer ratio of 72:28 (mol%) and measured by SEM (SU8020; Hitachi Co., Japan) operated at an accelerating voltage of 5 kV after ion sputtering (E-1045; Hitachi Co., Japan) at 10 mA. (d) Hydrodynamic diameter of the OTN-PSt NPs with different mixture ratio of styrene and acrylic acid monomers. The diameters were determined by dynamic light scattering as shown in Materials and Methods. The diameters of the size distribution peak of each sample were also shown in Table 1.

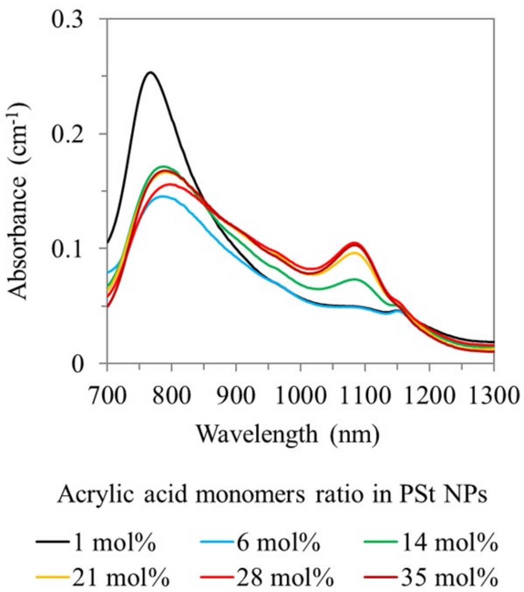

Supplementary Figure S2. Absorption spectra of OTN-PSt NPs (5 mg/mL in water) with different ratio of styrene and acrylic acid monomers. The absorption peak of 1050-1150 nm is majorly luminescent IR-1061, while the absorption of 750-800 nm is derived from IR-1061 interacted with water. Adding acrylic-acid monomer increased the loading of emissive IR-1061 to OTN-PSt NPs; however, IR-1061 seemed to interact strongly with water when the NPs were prepared in the solvent containing 5% DMSO.

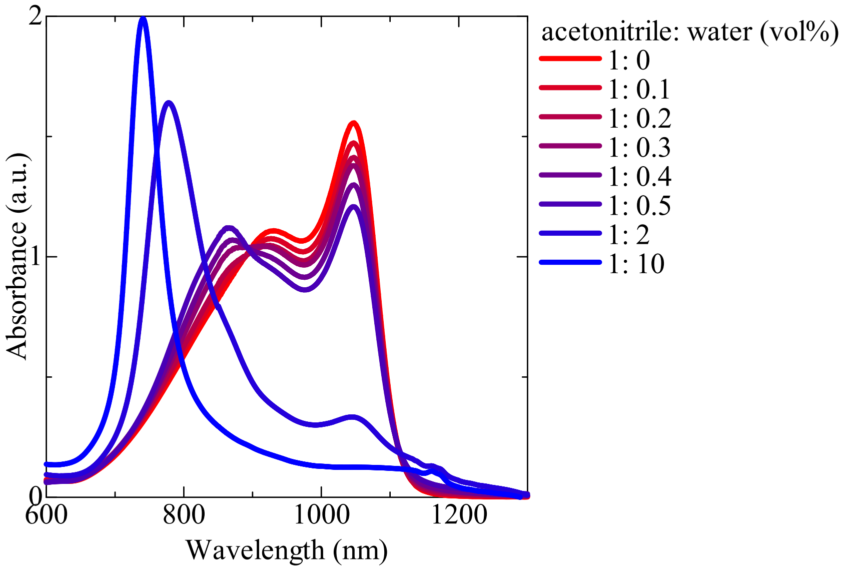

Supplementary Figure S3. Effect of water contamination on the absorption spectrum of IR-1061 dissolved in acetonitrile. The optical absorption was analyzed for IR-1061 solution (12.5 μg/mL) in each solvent.

Analysis of quantum yield of the OTN-PSt-PEG NP

The fluorescence quantum yield of OTN-PSt-PEG NPs in water was analyzed according to the previous publication(*1) using the OTN-NIR fluorescence IR-26 dye as the reference (quantum yield = 0.5%). For reference calibration, IR-26 dye was diluted by 1,2-dichloroethane (DCE; refractive index: 1.44) to prepare five samples with their absorbance value at 980 nm of ~0.14, ~0.12, ~0.10, ~0.08, and ~0.06. Then, the fluorescence emission was collected in the 1000-1500 nm region under an extinction wavelength of 980 nm. The similar operation were also performed for OTN-PSt-PEG NPs in water (refractive index: 1.33). All of the emission profiles of samples were integrated into the 1000-1500 nm region, and the integrated photoluminescence was plotted against the absorbance intensity (980 nm) to obtain a linear function. Finally, the fluorescence quantum yield was calculated based on the equation as follows:

$$QY_{sample} = QY_{ref} \cdot (slope_{sample} / slope_{ref}) \cdot (n_{sample} / n_{ref})^2$$

where $slope_{sample}$ and $slope_{ref}$ are the slopes of the linear equation fitted from OTN-PSt NPs and IR-26, respectively, n_{sample} and n_{ref} are the refractive indices of water and DCE, respectively.

(*1) Guosong Hong, Yingping Zou, Alexander L. Antaris, Shuo Diao, Di Wu, Kai Cheng, Xiaodong Zhang, Changxin Chen, Bo Liu, Yuehui He, Justin Z. Wu, Jun Yuan, Bo Zhang, Zhimin Tao, Chihiro Fukunaga and Hongjie Dai, Ultrafast fluorescence imaging *in vivo* with conjugated polymer fluorophores in the second near-infrared window, *Nat. Commun.*, 2014, **5**, 4206.

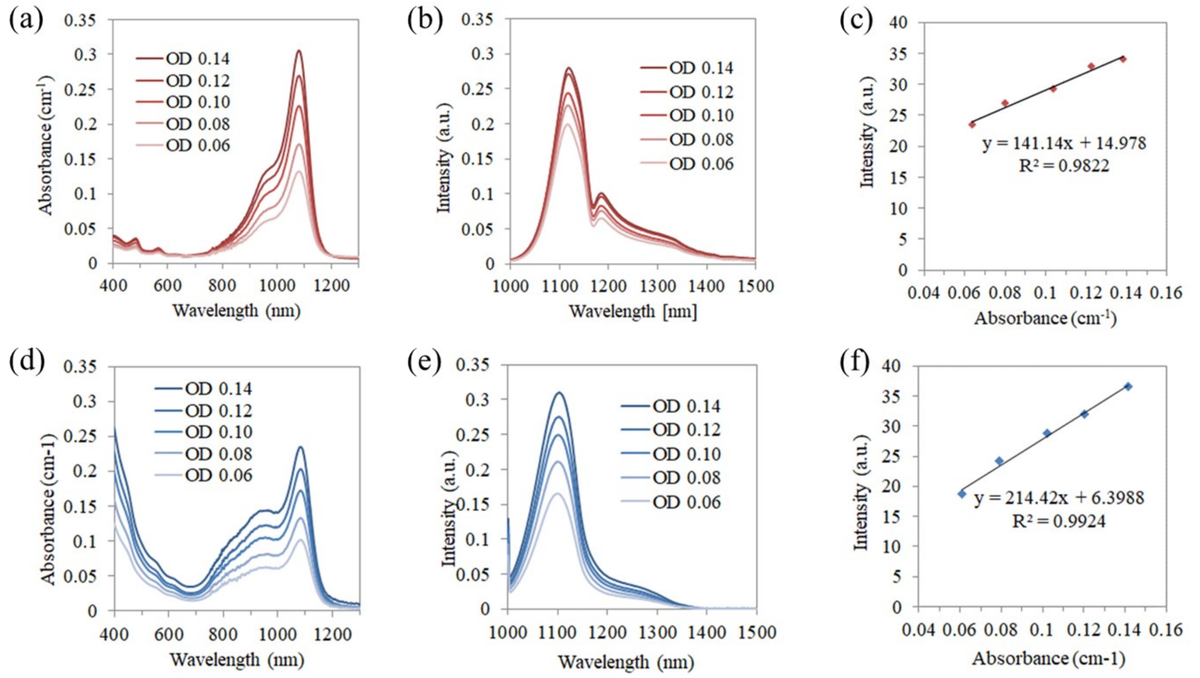

Supplementary Figure S4. Quantum yield measurement.
(a) UV-vis-NIR absorption spectra of a series of the IR-26 reference solutions in DCE with increasing concentrations. (b) Photoluminescent spectra of the IR-26 reference solutions shown in (a) under excitation of 980 nm. (c) Integrated OTN-NIR photoluminescent intensity plotted as a function of absorbance at 980 nm for IR-26 reference solutions based on the measurements of (a) and (b). (d) UV-Vis-NIR absorption spectra of a series of the OTN-PSt-PEG NPs with a styrene to acrylic acid ratio of 72:28 (mol%) in water with increasing concentrations. (e) Photoluminescent spectra of the OTN-PSt-PEG NPs with a styrene to acrylic acid ratio of 72:28 (mol%) shown in (d) under an excitation of 980 nm. (f) Integrated OTN-NIR photoluminescent intensity plotted as a function of absorbance at 980 nm for OTN-PSt-PEG NPs with a styrene to acrylic acid ratio of 72:28 (mol%) in water based on the measurements of (d) and (e). The data was fitted into a linear function with a slope of 141.14, giving a measured quantum yield of 0.65%.
